# Cartilage Tissue Engineering Using Dermis Isolated Adult Stem Cells: The Use of Hypoxia during Expansion versus Chondrogenic Differentiation

**DOI:** 10.1371/journal.pone.0098570

**Published:** 2014-05-27

**Authors:** Kerem N. Kalpakci, Wendy E. Brown, Jerry C. Hu, Kyriacos A. Athanasiou

**Affiliations:** 1 Medtronic Spine & Biologics, Memphis, Tennessee, United States of America; 2 UC Davis, Department of Biomedical Engineering, Davis, California, United States of America; University of Pittsburgh, United States of America

## Abstract

Dermis isolated adult stem (DIAS) cells, a subpopulation of dermis cells capable of chondrogenic differentiation in the presence of cartilage extracellular matrix, are a promising source of autologous cells for tissue engineering. Hypoxia, through known mechanisms, has profound effects on *in vitro* chondrogenesis of mesenchymal stem cells and could be used to improve the expansion and differentiation processes for DIAS cells. The objective of this study was to build upon the mechanistic knowledge of hypoxia and translate it to tissue engineering applications to enhance chondrogenic differentiation of DIAS cells through exposure to hypoxic conditions (5% O_2_) during expansion and/or differentiation. DIAS cells were isolated and expanded in hypoxic (5% O_2_) or normoxic (20% O_2_) conditions, then differentiated for 2 weeks in micromass culture on chondroitin sulfate-coated surfaces in both environments. Monolayer cells were examined for proliferation rate and colony forming efficiency. Micromasses were assessed for cellular, biochemical, and histological properties. Differentiation in hypoxic conditions following normoxic expansion increased per cell production of collagen type II 2.3 fold and glycosaminoglycans 1.2 fold relative to continuous normoxic culture (*p*<0.0001). Groups expanded in hypoxia produced 51% more collagen and 23% more GAGs than those expanded in normoxia (*p*<0.0001). Hypoxia also limited cell proliferation in monolayer and in 3D culture. Collectively, these data show hypoxic differentiation following normoxic expansion significantly enhances chondrogenic differentiation of DIAS cells, improving the potential utility of these cells for cartilage engineering.

## Introduction

Hyaline articular cartilage and fibrocartilage of temporomandibular joint (TMJ) and knee meniscus lack the intrinsic ability for self-repair. Efforts to engineer tissues *in vitro* using primary cells have resulted in constructs with clinical dimensions and functional properties approaching those of native cartilage [1], [2] and fibrocartilage [3], [4]. As the isolation of primary cells for autologous treatments is associated with invasive procedures, limited cell availability, and donor site morbidity, a significant challenge on the path toward clinical translation of these efforts is the development of appropriate cell sources.

A variety of stem cells, both embryonic [5]–[8] and adult [9]–[11], have been explored for their chondrogenic potential. While stem cells have been isolated from numerous adult tissues, skin is a particularly attractive source as it is highly regenerative and minimizes the invasiveness of cell isolation. It has been shown that the dermis contains a population of cells capable of chondrogenesis [12]–[17]. Our group has found that adult dermal fibroblasts are capable of chondrogenic differentiation when cultured on the aggrecan, the main proteoglycan found in cartilage [13]. We showed that a subpopulation with high chondrogenic potential, termed dermis-isolated adult stem (DIAS) cells, could be selected through rapid adherence to tissue culture polystyrene surfaces [18], [19]. We also demonstrated the ability of DIAS cells to not only produce cartilage-like extracellular matrix (ECM), but also form tissue engineered constructs with robust mechanical properties [16]. However, the properties of constructs engineered using DIAS cells do not reach native tissue biochemical and biomechanical values; therefore it is necessary to further enhance the DIAS cell expansion and chondrodifferentiation protocols.

Mesenchymal stem cells (MSCs) are sensitive to ambient oxygen levels during *in vitro expansion* [20]. Reduced oxygen tension (<5% O_2_) has been shown to increase proliferation [21], enhance colony-forming efficiency [22], and prevent phenotypic drift [23] during monolayer culture of bone-marrow derived MSCs. In addition, hypoxic (2% O_2_) expansion of adipose-derived stem cells (ASCs) upregulates collagen II gene expression and increases sulfated glycosaminoglycan (GAG) deposition in high density culture relative to normoxic (21% O_2_) expansion, suggesting that hypoxia may influence early chondrogenesis [24]. In dermal fibroblasts, reduced oxygen tension during monolayer culture modulates expression of a myriad proteins, including those related to transcriptional control, metabolism, and matrix remodeling [25], though the effect of hypoxia during expansion on subsequent chondrogenic capacity of dermis cells is unknown.

MSC differentiation into chondrocytes during development occurs in a hypoxic environment (1–6% O_2_) [26], [27], and, *in vitro*, hypoxia promotes chondrogenic differentiation. Reduced oxygen tension during *differentiation* of stem cells upregulates expression of cartilage-related genes [8], [28]–[31] and increases production of cartilage-specific matrix [28], [31]–[33]. A study by Mizuno *et al*. [34] showed enhanced chondrogenesis of immature dermal fibroblasts seeded on collagen/demineralized bone powder sponges in low oxygen (5% O_2_), and increased production of hypoxia inducible factor-1α (HIF-1α), a known regulator of hypoxic chondrogenic differentiation [35]. Others have elucidated the mechanism of hypoxia-induced chondrogenic differentiation in stem cells [26], [36]–[38], therefore it is the aim of this study to characterize the functional outcomes of DIAS cell exposure to hypoxia. Based on these findings, it was hypothesized that the chondrogenic differentiation of DIAS cells would be enhanced under low ambient oxygen.

In an effort to refine the expansion and differentiation processes for DIAS cells, and to gain an understanding of how hypoxia affects the chondrogenesis of DIAS cells, this study examined the effects of hypoxia during expansion and chondrogenic differentiation. We hypothesized that exposure to hypoxia during expansion and/or differentiation would enhance chondrogenesis of DIAS cells. The primary criterion for evaluating chondrogenesis was collagen type II production during differentiation, both overall and relative to cell number and total collagen production. Sulfated GAG production was also quantified, and deposition of collagen types I and II, total collagen, and GAGs were examined histologically. In addition, we examined proliferation and colony forming units (CFU-F) in monolayer to evaluate the effects of hypoxia on the cell-growth characteristics of DIAS cells.

## Methods

### Dermal Fibroblast Isolation

Full-thickness skins from the abdomens of seven adult goats were obtained from a local abattoir (Fisher Ham & Meat, Spring, TX). The dermis was isolated, minced, and digested in medium containing 0.2% type II collagenase (Worthington, Lakewood, NJ) at 37°C with agitation. Base medium consisted of DMEM with 4.5 g/L glucose and L-glutamine (Gibco, Grand Island, NY), 1% penicillin/streptomycin/fungizone (Biowhittaker, Walkersville, MD), and 1% non-essential amino acids (Life Technologies, Gaithersburg, MD). Digests were diluted with expansion medium (base medium with 10% FBS [Biowhittaker]), filtered, and centrifuged at 300 *g*. Cells were resuspended in expansion medium, combined, and plated in flasks.

### Expansion in Hypoxia and Normoxia and Isolation of a Chondroinducible DIAS Cell Subpopulation

Cells were cultured separately in hypoxic (5% O_2_) or normoxic (20% O_2_) incubators. All liquids were preconditioned in 100 mm petri dishes in the respective incubators for >12 h prior to use on cell cultures to acclimate to environmental O_2_ levels [39]. Upon reaching 80–85% confluence (∼5 days), cultures were treated with 0.5% Dispase (BD, Franklin Lakes, NJ) for 15 minutes to detach keratinocytes, and the non-adherent cells were discarded. After expansion to 80–85% confluence (∼3 days), all cells were lifted using trypsin and EDTA (Sigma, St. Louis, MO). To isolate the chondroinducible subpopulation (DIAS cells), the cell suspension was exposed to tissue-culture polystyrene (TCP) flasks for 10 minutes, and floating cells were discarded [18]. The flasks were washed 3x with PBS and cultured in expansion medium until reaching 80–85% confluence (∼5 days), then lifted using trypsin and EDTA for monolayer assays and differentiation culture.

### Assessment of Monolayer Proliferation and Clonogenesis

DIAS cells were resuspended and 10^4^ cells were added to 100 mm petri dishes with expansion medium and cultured in hypoxic (5% O_2_) or normoxic (20% O_2_) incubators. At each time point (day 0, 1, 3, 5, 7, 9, and 11), cells were collected and frozen at −80°C. After collecting cells from each time point, cell number was determined using Picogreen Cell Proliferation Assay Kit (Molecular Probes, Eugene, OR).

For DIAS cell CFU (CFU-F) assessment, 10^2^ DIAS cells were cultured undisturbed in 100 mm Petri dishes for 2 weeks. Dishes were fixed with methanol, stained with 2% crystal violet, and colonies greater than 2 mm diameter were counted. The % CFU-F was determined as the number of colonies normalized to seeded cells. Due to limitations in cell number and processing, cells used for clonal analysis were not used for chondrogenic differentiation.

### Chondrogenic Differentiation on Coated Surfaces

Cells expanded separately in monolayer from those used in clonal analysis were cultured in both environments during differentiation (hypoxia → hypoxia (HH), hypoxia → normoxia (HN), normoxia → hypoxia (NH), normoxia → normoxia (NN)). The micromass differentiation protocol was modified from the procedure described by Ahrens *et al*. [40]. This method was shown to induce DIAS cell chondrogenesis in a manner similar to the aggrecan method described previously [18] in an unpublished study from our laboratory. Coated surfaces were prepared in 24-well TCP plates. A sterile 0.08% chondroitin sulfate (Sigma) solution was prepared and 20 µl was dropped into each well and allowed to dry overnight.

DIAS cells were suspended in chondrogenic medium consisting of base medium with 50 µg/ml ascorbic acid-2-phosphate (Acros Organics, Geel, Belgium), 0.4 mM proline (Acros), 50 mg/ml ITS+ Premix (BD Biosciences, Bedford, MA), 10^−7^ M dexamethasone (Sigma), 10 ng/ml transforming growth factor β1 (TGF-β1) (Peprotech, Rocky Hill, NJ), 100 ng/ml recombinant human insulin-like growth factor (Peprotech), and 1% FBS. 2×10^5^ cells were seeded in a 20 µl droplet on the dried surface. After 4 hours, 500 µl of chondrogenic medium was carefully added around the condensed cell mass, and 250 µl of media was exchanged every other day for 14 days.

### Quantitative Biochemistry

After 14 days, the contents of four wells were combined to make one sample for quantitative biochemical analysis. Samples were digested in pepsin (10 mg/ml) with acetic acid, followed by pancreatic elastase (1 mg/ml) in Tris buffer at 4°C. Cellularity was determined using the Picogreen kit. Total sulfated GAG content was determined using a dimethylmethylene blue (DMMB) dye-binding assay kit (Biocolor, Newtownabbey, Northern Ireland). Total collagen content was determined after hydrolyzing samples with 2 normal NaOH for 20 minutes at 110°C with a chloramine-T hydroxyproline assay using Sircol standards (Biocolor) [41].

Collagen type II was quantified using an indirect enzyme-linked immunosorbent assay (ELISA). Samples and standards were incubated in a 96-well plate overnight at 4°C. Wells were blocked with BSA overnight at 4°C, then exposed to a primary antibody, anti-collagen type II IgG (Cedarlane Labs, Burlington, NC) for 1 hour at 20°C. The secondary antibody, anti-IgG horseradish peroxidase (Millipore, Temecula, CA) was then applied for 1 hour at 20°C. Between each incubation step, wells were washed 3x with 0.05% Tween-20. Results were visualized at 450 nm using a TMB substrate.

### Histology and Immunohistochemistry

Micromasses were cryo-sectioned at 14 µm. Histology sections were fixed in 10% phosphate buffered formalin and stained with picrosirius red to examine collagen. Immunohistochemical (IHC) analyses for collagen types I and II were performed on acetone-fixed sections using primary antibodies from US Biological (anti-collagen type I, Swampsacott, MA) and Cedarlane Labs (anti-collagen type II). Secondary antibodies and avidin-biotinylnated enzymes (VectastainABC kit, Burlingame, CA) were applied, followed by DAB reagent (Vector labs), and slides were counterstained with hematoxylin.

### Statistical Analyses

For proliferation assays, n = 3 was used at each time point, while n = 5 was used for CFU-F and quantitative biochemistry. Data were analyzed with a two-factor analysis of variance (ANOVA), using Tukey's HSD *post hoc* where applicable. Significance was defined as *p*<0.05, and data are reported as mean ± standard deviation.

## Results

### Cell Growth and CFU-F

**Figure 1:**
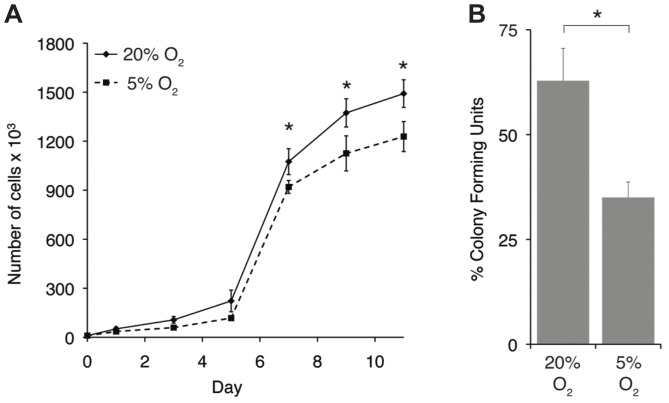
Proliferation and clonogenesis of cells in monolayer. (A) Cells proliferated faster in normoxia than hypoxia. Cell number was greater in normoxia at days 7 (*p* = 0.04), 9 (*p* = 0.04), and 11 (*p* = 0.002). (B) CFU-F was greater in normoxia than hypoxia (*p*<0.0001). Data are mean ± S.D.

The examination of cell proliferation revealed differences in cell growth kinetics in hypoxic and normoxic culture (Fig. 1A). There was no statistical difference between the groups over the first 5 days of growth, though normoxic culture resulted in a statistically significant increase in cell number at days 7, 9, and 11, compared with hypoxic culture. Cells cultured in normoxia also had a higher CFU-F (63±8%) than hypoxic cultured cells (35±4%) (Fig. 1B).

### Cellularity of Micromass Cultures

**Figure 2:**
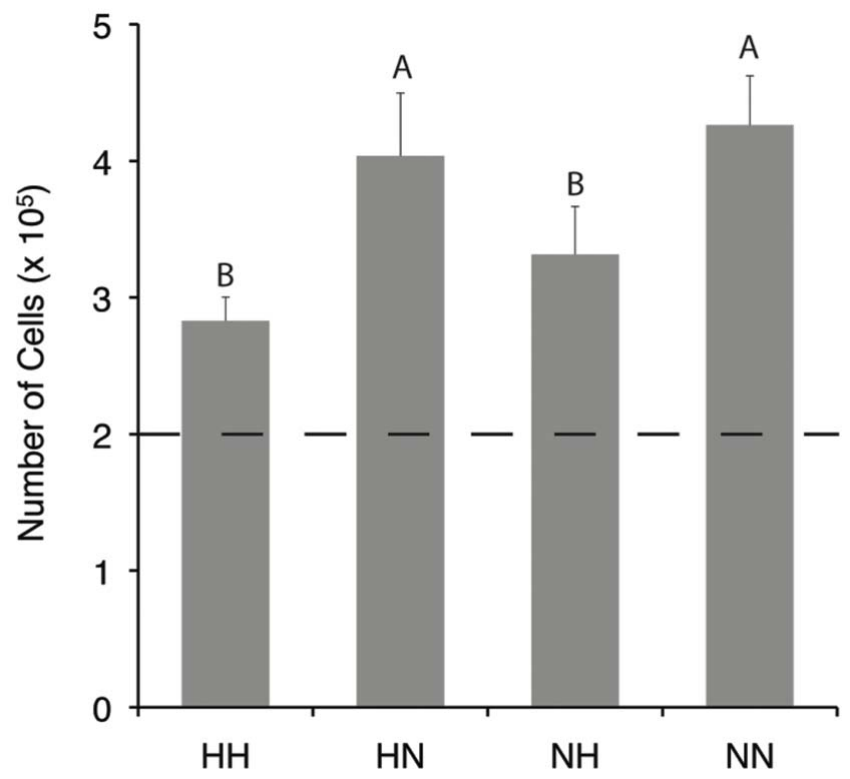
Hypoxia limits proliferation during 3D culture. Cells were seeded at 2×10^5^ per micromass (dashed line). Data are mean ± S.D. Groups not connected by letters are significantly different with *p*<0.05.

Cellularity increased during the 14 days of 3D culture for all treatments (Fig. 2). Hypoxic differentiation decreased cell growth 26% (*p*<0.0001) relative to normoxic differentiation, while hypoxic expansion led to a 9% decrease (*p* = 0.04) in cellularity of micromasses relative to normoxic expansion. There were no statistical differences between the NN and HN groups (*p* = 0.8), or NH and HH groups (*p* = 0.2).

### Total Collagen Production

**Figure 3:**
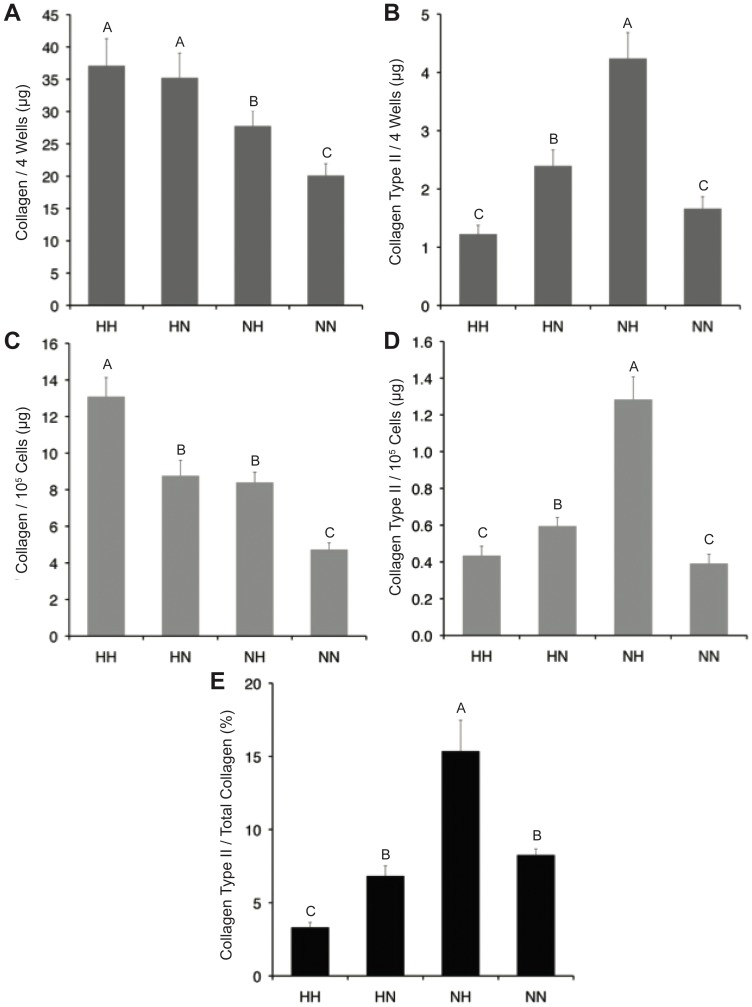
Collagen accumulation during micromass culture. Hypoxic expansion led to increased total collagen accumulation (A) and collagen accumulation per cell (C) during differentiation. Overall collagen type II production was greatest in the NH group (B) as was collagen type II production per cell (D) and relative to total collagen production (E). Data are mean ± S.D. Groups not connected by letters are significantly different. Significance defined as *p*<0.05.

Data for total collagen production during differentiation are shown in Figs. 3A and 3C. Cells expanded in hypoxia produced 51% more total collagen than normoxia-expanded cells (*p*<0.0001), while hypoxic differentiation increased collagen production 17% (*p* = 0.004). Hypoxic differentiation also led to an increase in collagen production of 59% (*p*<0.0001) relative to normoxic differentiation, normalized to 10^5^ cells.

### Collagen Type II Production

Data for collagen type II production during differentiation are shown in Figs. 3B and 3D. Hypoxic expansion decreased total production of collagen type II 39% and per 10^5^ cell production 38%, while hypoxic differentiation increased total production of collagen type II 62% and per 10^5^ cell production 85% (*p*<0.0001 for all comparisons). The NH group produced 1.6 fold more collagen type II (*p*<0.0001) and 2.3 fold more collagen type II per 10^5^ cell (*p*<0.0001) than the NN group. HN micromasses contained 95% more collagen type II (*p*<0.0001) and 37% more collagen type II per 10^5^ cell (*p* = 0.02) than NN micromasses. Collagen type II/total collagen (Fig. 3E) was 1.3 fold greater for groups expanded in normoxia (NN, NH) relative to hypoxic expansion (*p*<0.0001) and the ratio was increased 23% (*p* = 0.003) with subsequent hypoxic differentiation. The NH group had a collagen type II/total collagen ratio of 0.15, statistically greater than all other groups.

### GAG Production

**Figure 4:**
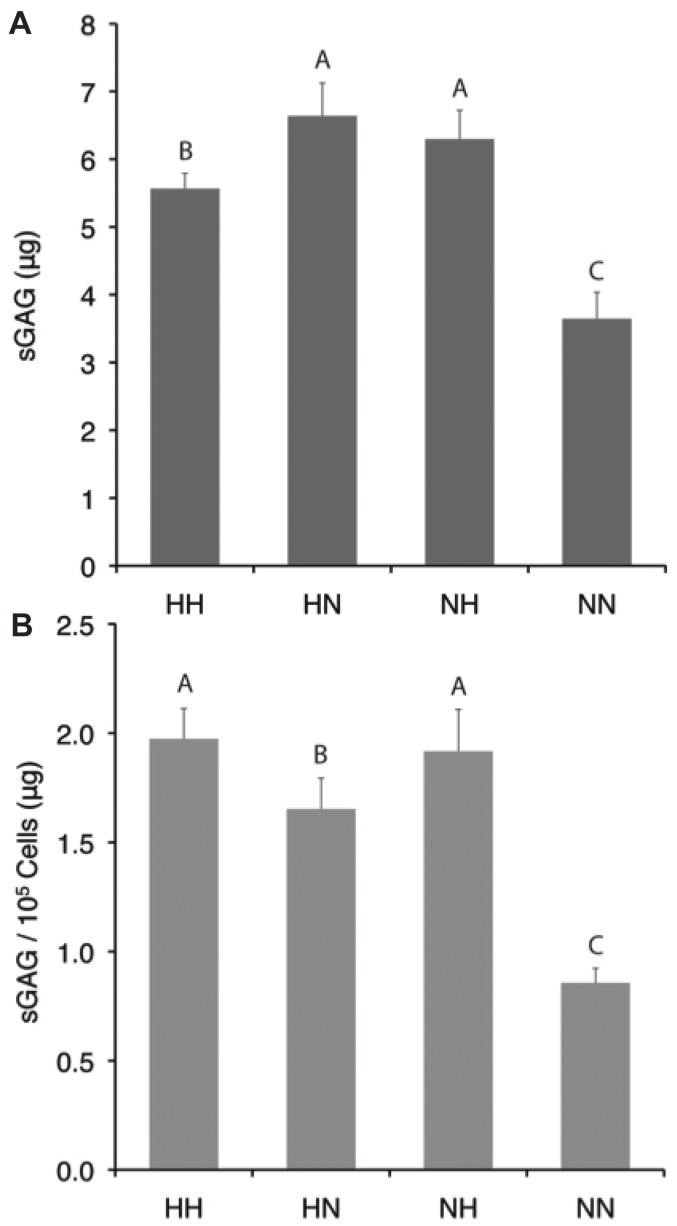
GAG accumulation during micromass culture. Cells exposed to hypoxia (HH, HN, NH) produced more GAG overall (A) and per cell (B) relative to continuous normoxic culture (NN) (*p*<0.05). Data are mean ± S.D. Groups not connected by letters are significantly different.

Data for sulfated GAG content are shown in Fig. 4. Hypoxic expansion increased total sulfated GAG production by 23% (*p*<0.0001), while hypoxic differentiation increased GAGs by 15% (*p*<0.0001). Similar trends were apparent when normalized to cell number. For normoxia-expanded groups, hypoxic differentiation (NH) increased GAG by 73% and increased GAG per cell by 124% over normoxic differentiation (NN) (*p*<0.0001). For the hypoxia-expanded groups, hypoxic differentiation (HH) decreased total GAG by 16% (*p*<0.0001) but increased per cell GAG production by 19% (*p* = 0.01) over normoxic differentiation (HN).

### Histological Evaluation

**Figure 5:**
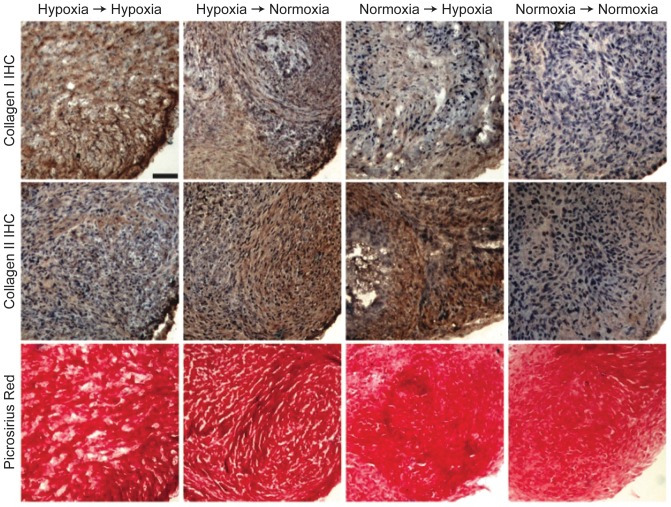
Histological sections of micromasses after 2 weeks. Collagen type I staining was strongest for HH (first row), while collagen type II staining was strongest for NH (second row). All groups stained positive for collagen (third row).

Representative pictures from the histological examination of micromass ECM from all groups are presented in Fig. 5. Immunostaining revealed the presence of collagen types I and II in all constructs. Collagen type I staining was strongest in the HH group, and weakest in the NN group. Collagen type II staining was more prominent in the NH group, while staining in the NN and HH groups was less evident. All groups stained brightly for collagen with picrosirius red with no apparent differences among groups.

## Discussion

The overall aim of this study was to enhance *in vitro* chondrogenesis of DIAS cells by examining the effects of reduced oxygen tension. Cells were cultured in hypoxic (5% O_2_) and normoxic (20% O_2_) environments during expansion and differentiation, and chondrogenesis was assessed through analysis of ECM production and cell-growth characteristics. Hypoxic *differentiation* (NH) dramatically increased production of collagen type II and increased the ratio of collagen type II to total collagen production compared to continuous normoxic culture (NN), Hypoxic differentiation (NH) also dramatically increased sulfated GAG production per cell compared to continuous normoxic exposure (NN). These results together identify NH as the best treatment to enhance chondrogenesis of DIAS cells. In addition, hypoxic *expansion* (HN and HH) significantly increased total matrix production relative to continuous normoxic culture (NN). These results demonstrate the potent effects of oxygen tension on the chondrogenic ability of DIAS cells, and improve the utility of these cells for cartilage and fibrocartilage tissue engineering.

In general, chondrogenic differentiation of skin-derived progenitor cells has been evaluated via histology and immunofluorescence [17], [42], [43], making this study is the first to quantify collagen type II protein production by DIAS cells. The group that produced the greatest amount (NH) synthesized 1.3 µg of collagen type II per 10^5^ cells, on par with reported values for ASC chondrogenesis at 2 weeks [44]. Collagen type II is produced in significant quantities almost exclusively in cartilaginous tissues. It is the most abundant protein in hyaline articular cartilage, while fibrocartilages from the TMJ and knee meniscus contain varying ratios of collagen type II to other collagens [45]. The production of this protein in comparison with other matrix constituents can provide a quantitative measure of the chondrocytic character of cells following chondrogenic treatments. In this study, 15% of the total collagen produced by the NH group was type II, compared to 8% for the NN and HN groups and 3% for the HH group. These values are all indicative of a fibrochondrocyte phenotype, though hypoxic differentiation advanced the cells further towards a chondrocyte phenotype.

In this study, we also examined the effect of hypoxic expansion on subsequent chondrogenesis and explored whether pre-differentiation exposure to hypoxia would select for or ‘prime’ the chondrogenic subpopulation. While this has been shown for ASCs [24], this study does not conclusively show that such is also the case for DIAS cells. With respect to engineering cartilage, there have been conflicting results pertaining to the application of hypoxia and its benefits and drawbacks [24], [46]–[49]. It was recently shown that hypoxia applied during the collagen synthesis phase versus the collagen maturation phase of a developing tissue has drastically different outcomes [50]. A similar temporal dependence for hypoxia's use in expansion and differentiation likely exists and should be further explored in future studies. Continuous hypoxic exposure (HH) increased overall matrix production but reduced the collagen type II to total collagen ratio. Coupled with the monolayer expansion assessments that showed decreased proliferation and clonogenesis, the results indicate a phenotypic switch from mitotic to biosynthetic activity. The possible presence of non-chondrogenically differentiated, mature fibroblasts in the system does not likely contribute to the decrease in proliferative activity of DIAS cells under hypoxia because while monolayer culture of fibroblasts under chronic hypoxia is known to upregulate expression of TGF-β1 [51], and α1(I) procollagen [52], [53], increase synthesis of collagen type I [54], and limit cell proliferation [55], [56], acute (less than six passages) exposure to hypoxia is known to increase fibroblast population doublings [57]. From these data, we can speculate that exposure of cells to hypoxia during monolayer expansion limited the chondrogenic ability of DIAS cells prior to differentiation in 3D by reducing cell plasticity and committing cells to a synthetic phenotype. Interestingly, the collagen type II per total collagen ratio of the HN was unchanged relative to the NN group, indicating (at least partial) reversal of this response upon reoxygenation, a phenomenon supported by the literature [56].

The mechanism of hypoxia in *in vitro* chondrogenic differentiation of stem cells is established. For example, in hypoxic culture of MSCs, prolyl hydroxylase activity is reduced, allowing the HIF-1α subunit of the HIF dimer to stabilize and translocate to the nucleus where it accumulates [37]. Acting in concert with HIF-1β, HIF-1α binds to hypoxia responsive elements, thereby initiating the transcription of genes involved in chondrogenesis and cartilage-specific extracellular matrix production such as collagen II, lysyl oxidase, chondroitin-4-sulfonotransferase-2, and Sox9 [26], [37], [38]. Furthermore, hypoxia-induced increases in collagen II and Sox9 expression and proteoglycan deposition are inhibited by HIF-1α siRNA [37]. The *in-vitro* effects of hypoxia mirror the role of hypoxia in developmental chondrogenesis which takes place in an environment of 1–6% O_2_ [27], [58]. The role of hypoxia in developmental chondrogenesis and the chondrogenic differentiation of established stem cells, such as bone marrow-derived MSCs and adipose derived stem cells, is well-known and convergent [36]. While further studies should be conducted to confirm the same mechanisms in DIAS cells, this knowledge and its conservation between cell types shifts importance towards characterizing the functional effects of hypoxia.

Methodologies that facilitate *in vitro* stem cell chondrogenesis typically have a basis in physiological processes or characteristics of native cartilage. In this and our previous study [18], DIAS cells were grown on physiological surfaces; the cartilage-derived matrix molecule aggrecan, and its predominant GAG, chondroitin sulfate. These surfaces induce a physiological response in which cells assemble into high-density aggregates, reminiscent of mesodermal cell condensation which precedes nascent cartilage development *in utero* [59]. The reduced oxygen level utilized in this study is similar to that experienced by native chondrocytes, estimated to be 1–8% depending on tissue location [60], [61]. In this study, concurrent application of signals derived from native cartilage physiology, comprising high-density cell culture, cell-matrix interactions, soluble chondrogenic agents, and reduced oxygen tension, enhanced chondrogenesis. Exploration into temporal application of these and additional stimuli, motivated by native cartilage physiology, will likely enhance this process further.

In conclusion, this study demonstrates enhancement of DIAS cell chondrogenesis by applying hypoxic culture conditions. Translation of *in vitro* cartilage regeneration models will require identification of clinically useful cells, and the use of skin as a donor tissue is especially promising due to the ease of procurement and negligible damage to the donor site. The final goal of this work is to use patients' own skin cells to create functional, autologous tissues for cartilage repair and replacement. Building on our previous study, which demonstrated the isolation and differentiation of a chondroinducible subpopulation of cells from the dermis, and these findings provide an important advancement towards that goal.
